# Sore Throat, Fever, Septic Emboli, and Acute Respiratory Distress Syndrome: A Case of Lemierre Syndrome

**DOI:** 10.1155/2018/7373914

**Published:** 2018-12-06

**Authors:** Farrukh N. Jafri, Jodi Shulman, José C. Gómez-Márquez, Matthew Lazarus, David M. Ginsburg

**Affiliations:** ^1^M.D., Assistant Director of Education and Simulation, Assistant Professor of Emergency Medicine at the Albert Einstein College of Medicine, Department of Emergency Medicine, White Plains Hospital, White Plains, NY, USA; ^2^PA-C, Department of Emergency Medicine, White Plains Hospital, White Plains, NY, USA; ^3^M.D., Department of Intensive Care Unit, White Plains Hospital, White Plains, NY, USA; ^4^M.D., Department of Radiology, Montefiore Medical Center, Bronx, NY, USA

## Abstract

Lemierre's syndrome is an acute oropharyngeal infection with a secondary septic thrombophlebitis of the internal jugular vein (IVJ) that was first reported in literature in 1936. It involves the progression of disease from a focal suppurative peritonsillar infection to a local septic thrombophlebitis with hematogenous progression to and distant septic emboli. It is a rare and potentially fatal syndrome requiring prompt diagnosis and management. We present the case progression of an 18-year-old male who presented to our hospital with resolved sore throat, fever, and chest discomfort who experienced a sharp clinical decline. His case, physical exam, laboratory abnormalities, and radiologic studies highlight important facets of this rare but important syndrome.

## 1. Case Description

An eighteen-year-old male presented to the Emergency Department with fever and severe chest pain. His symptoms developed five days earlier when he woke up with a sore throat. He was seen by his pediatrician who performed a negative Rapid Group-A Streptococcus swab. Over the following three days, he developed nausea, vomiting, intermittent fevers, sweats, and chills. He went back to his pediatrician who this time started him empirically on oseltamivir phosphate for presumed influenza. His symptoms of sore throat and chills improved the night prior to admission, but then he developed severe suprasternal chest discomfort and pleuritic chest pain. He had never used alcohol or illicit drugs.

**Figure 1:**
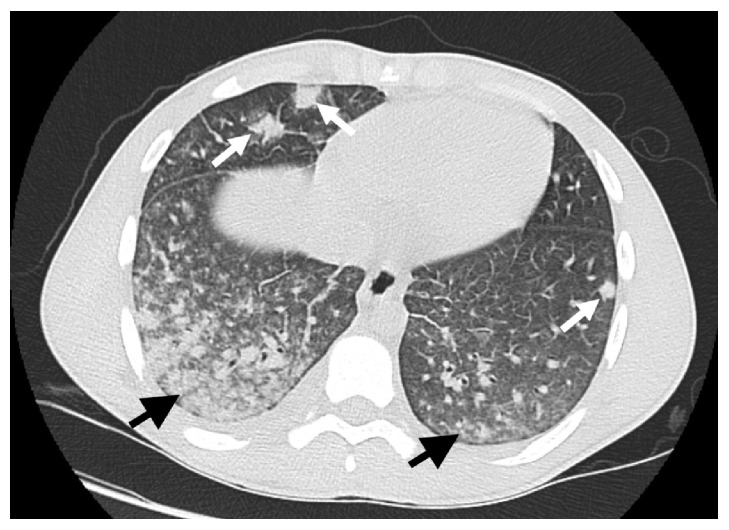
Axial image from CT scan of the chest without intravenous contrast. There are bibasilar patchy infiltrates, right greater than left (black arrows). There are additional scattered nodular opacities (white arrows).

In the Emergency Department, the patient was initially afebrile, but shortly after his initial evaluation he developed a fever of 103 degrees Fahrenheit. He was hypotensive with a blood pressure of 88/46 mmHg and a heart rate of 127 beats per minute. His initial saturation was 99% on room air. On examination, he was noted to be drowsy but oriented to person, place, and time. He had moist mucous membranes and no throat exudates. He had no carotid bruits or cervical or axillary lymphadenopathy. His pulmonary auscultation was normal, and his heart exam had no murmurs. He had no cyanosis or mottling of skin. Labs were notable for white count of 17 thousand per mL with 28% bands, total bilirubin of 2.4 mg/dL, platelet count of 50 thousand per mL, lactic acid of 3.1 mg/dL, and a negative troponin. A plain chest X-ray had no acute abnormalities, and a noncontrast CT scan of his chest demonstrated bibasilar patchy infiltrates (Figure 1). After blood cultures were drawn and with a presumed diagnosis of bilateral community acquired pneumonia, Levofloxacin, and Clindamycin were initiated. Because of hypotension, bandemia, and lactic acidosis, he was admitted to the Intensive Care Unit for further monitoring. Within 12 hours, he had a rapid and sharp clinical decline with worsening and severe hypoxemia and marked progression of his bilateral pulmonary infiltrates readily evidenced on CXR. He required mechanical ventilation and was diagnosed with Acute Respiratory Distress Syndrome (ARDS). Pressure control ventilation with positive end-expiratory pressure (PEEP) up to 20 cm H20 and paralytics were needed to maintain adequate oxygenation and saturation. He was subsequently transferred to a tertiary care center for further management and possible Extracorporeal Membrane Oxygenation (ECMO) which in the end he did not require. Two days later, patient had two blood cultures which grew* Fusobacterium necrophorum*.

## 2. Diagnosis

**Figure 2:**
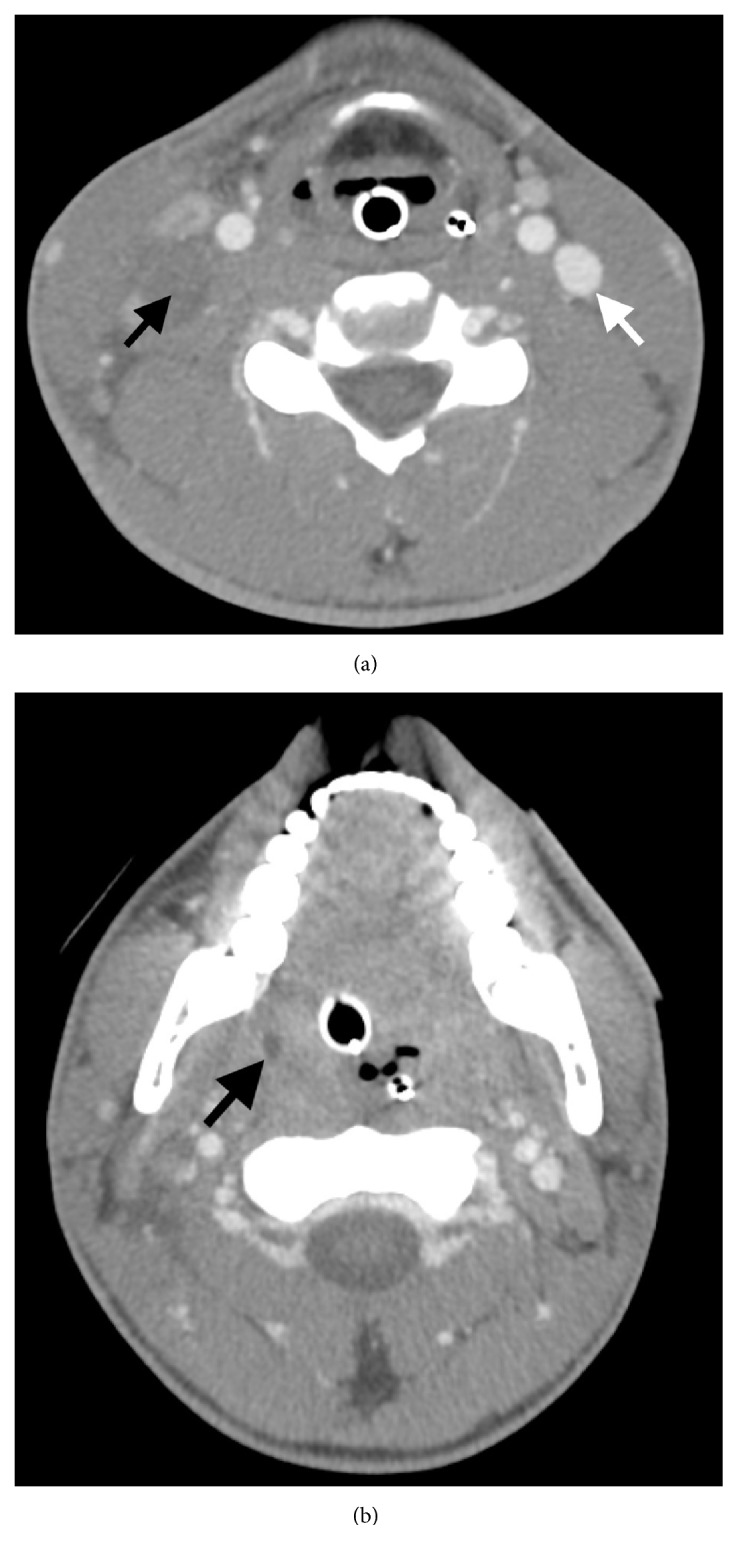
Axial image from CT scan of the neck with intravenous contrast. (a) There is complete occlusion of the right internal jugular vein (black arrow). The left internal jugular vein is patent (white arrow). A small amount of hypodense fluid surrounding the occluded right internal jugular vein reflects inflammatory changes. (b) Peritonsillar hypodensity (black arrow) measured up to 0.6 cm and was presumed to be a small abscess. Endotracheal and enteric tubes are noted.

**Figure 3:**
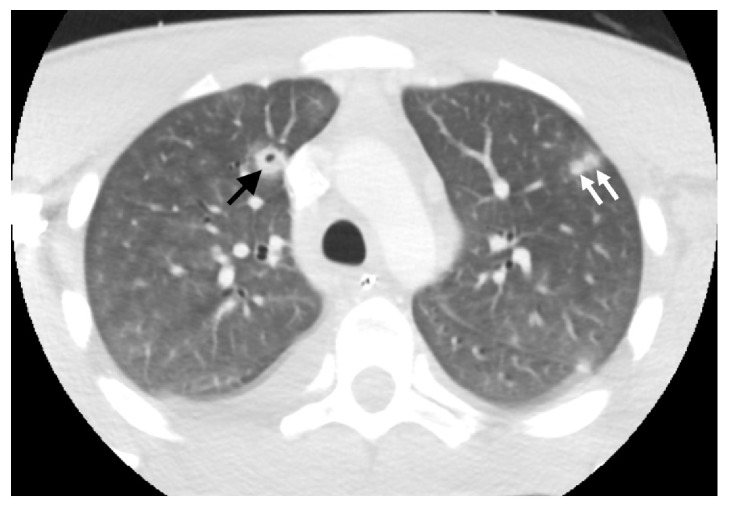
Axial image through the upper chest from CT scan of the neck. Multiple nodules were visualized in the imaged portion of the chest, including cavitary (black arrow) and noncavitary nodules (white arrows).

**Figure 4:**
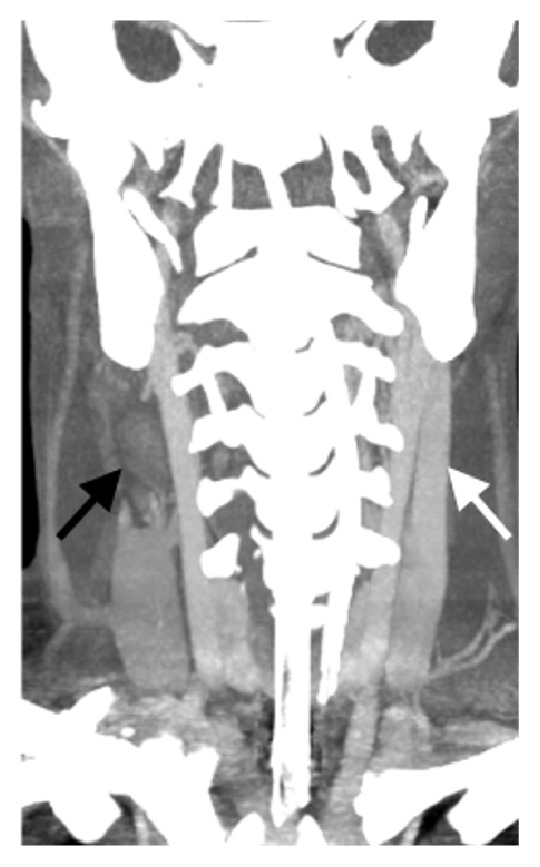
Coronal maximum intensity projection from CT scan of the neck with intravenous contrast. There is complete occlusion of the right internal jugular vein (black arrow). The left internal jugular vein is patent (white arrow).

Upon notification of the positive blood cultures, he had a CT scan of his neck with intravenous contrast of his neck and chest (Figures 2 and 3), demonstrating a right internal jugular thrombophlebitis, a 0.6 cm right peritonsillar abscess, and multiple pulmonary cavitary infiltrates consistent with septic emboli (Figure 4). Over the course of the next few days, he continued treatment with Meropenem and Linezolid and was liberated from mechanical ventilation. He was diagnosed with Lemierre's syndrome.

## 3. Discussion

Lemierre's syndrome (also called necrobacillosis or postanginal septicemia) is an acute oropharyngeal infection with a secondary septic thrombophlebitis of the internal jugular vein (IVJ). It was first reported by Courmont and Cade in 1900 but characterized in more detail by Lemierre in 1936 in a review of 20 cases [1]. Dr. Lemierre illustrated the progression of the disease from a focal suppurative peritonsillar infection to a local septic thrombophlebitis with hematogenous progression to and distant septic emboli [2]. The epidemiology of this condition is confounded by its rarity, conflicting definitions, and multiple aliases. A prospective study in Denmark reported an annual incidence of 3.6 per million per year [3], with the median age being 19 years. 89% of patients are between the ages of 10-35 years [4]. Despite its rarity, clinical experience suggests that its incidence may be on the rise, possibly secondary to antibiotic resistance, changes in prescription patterns, or the increased efficiency of diagnostic technologies [5].

*Fusobacterium necrophorum*, a normal inhabitant of the oral cavity, is the most common pathogen isolated in Lemierre's syndrome [1].* Fusobacterium necrophorum* is an anaerobic Gram-negative bacillus that has the ability to invade as a primary pathogen in previously healthy individual, a feature likely related to having a lipopolysaccharide endotoxin similar to aerobic Gram-negative bacilli [1]. The primary sources of infection tend to be the palatine tonsils and peritonsillar tissue, although pharyngitis, parotitis, otitis media, sinusitis, and mastoiditis have been described as causes. Infection proceeds from the oral cavity into the lateral pharyngeal space, which is divided into an anterior (muscular) and posterior (neurovascular) compartment. The carotid sheath and its components, the carotid artery and the internal jugular vein, are all found in the posterior compartment. An infection in this posterior compartment and particularly in the internal jugular vein as a thrombophlebitis can lead to metastatic infections via hematogenous bacterial dissemination, sepsis, and septic shock [1].

Clinically, the interval between oropharyngeal infection and onset of sepsis is usually a week or less [6]. If there is a lateral pharyngeal space infection, pain or swelling at the jaw or sternocleidomastoid may be present [7]. The thrombosed jugular vein is rarely palpated [1] and local findings can be subtle or absent (Golpe 16). The most common site of embolic disease is the lungs as was the case with our patient [6, 8].

The diagnosis of Lemierre's syndrome can be difficult and is often prompted by identification of* F. necrophorum* in blood cultures [2]. The diagnosis of this syndrome requires a high degree of clinical suspicion along with data that indicates IJV thrombophlebitis, sepsis, or septic emboli. A CT of the neck with contrast is the most useful investigation as it can reveal the vessels and adjacent soft tissue [1]. Ultrasound can also be used and, although less expensive and invasive, provides poor imaging beneath the clavicle and under the mandible and can miss fresh thrombus [8]. Magnetic resonance angiography can also be useful for diagnosing IJV thrombosis.

Due to the rarity of this disease, treatment recommendations are based on historical practice. The mainstay of treatment is intravenous antibiotics directed at anaerobic microbes as well as drainage of collections at primary or secondary sites of infection [1, 2]. With the advent of antibiotics, the mortality rate has dropped from 90% to 5% [2]. The role of anticoagulation of these patients is controversial, and the outcomes of patients appear to be good without it [1]. Prompt institution of anticoagulation may prevent thrombotic extension, embolization, and new thromboembolic events but may also expose patients to bleeding complications. It is also unclear whether anticoagulation may facilitate spread of septic material or result in a hemorrhagic transformation of embolic lesions [5]. When deciding on anticoagulation, the medical team must carefully consider the patients characteristics, severity of disease, bleeding risk, and risk of new thromboembolic complications as well as the drugs pharmacokinetics [5].

## 4. Conclusion

Lemierre's syndrome is a rare and potentially fatal syndrome requiring prompt diagnosis and management. Patients presenting with a history of an oropharyngeal infection with signs of sepsis as well as septic emboli should have consideration of this disease process, with a low threshold to evaluate for septic emboli as well as IJV thrombophlebitis.
